# Case report: Severe arrhythmogenic cardiomyopathy in a young girl with compound heterozygous *DSG2* and *MYBPC3* variants with a 6-year follow-up

**DOI:** 10.3389/fgene.2025.1545561

**Published:** 2025-03-06

**Authors:** Ryotaro Hashizume, Hiroshi Imai, Hiroyuki Ohashi, Hirofumi Sawada, Noriko Yodoya, Ryuji Okamoto, Kaoru Dohi, Chika Kasai, Takahito Kitajima, Takumi Fujiwara, Ikuyo Mochiki, Kaname Nakatani, Sachiko Wakita, Seiko Ohno, Koichi Kato, Yoshinaga Okugawa, Yoshihide Mitani, Masahiro Hirayama

**Affiliations:** ^1^ Department of Genomic Medicine, Mie University Hospital, Tsu, Mie, Japan; ^2^ Department of Pathology and Matrix Biology, Mie University Graduate School of Medicine, Tsu, Mie, Japan; ^3^ Pathology Division, Mie University Hospital, Tsu, Mie, Japan; ^4^ Department of Pediatrics, Mie University Graduate School of Medicine, Tsu, Mie, Japan; ^5^ Department of Cardiology and Nephrology, Mie University Graduate School of Medicine, Tsu, Mie, Japan; ^6^ Medical Genome Center, National Cerebral and Cardiovascular Center, Suita, Osaka, Japan; ^7^ Department of Cardiovascular Medicine, Shiga University of Medical Science, Otsu, Shiga, Japan

**Keywords:** arrhythmogenic cardiomyopathy, *DSG2*, compound heterozygous, *MYBPC3*, genetics, case report, clinical symptoms

## Abstract

**Introduction:**

Arrhythmogenic cardiomyopathy (ACM) is an inherited cardiac disorder characterized by progressive fibrofatty replacement of the myocardium. In the Japanese population, variants of the desmoglein-2 (*DSG2*) gene are a major cause of ACM, typically following an autosomal recessive inheritance pattern. Myosin-binding protein C (*MYBPC3*) variants are primarily associated with hypertrophic cardiomyopathy (HCM). Here, we report a severe pediatric case of ACM associated with compound heterozygous *DSG2* and *MYBPC3* variants.

**Case Presentation:**

A 6-year-old asymptomatic girl was diagnosed with ACM based on abnormal electrocardiogram findings, including epsilon waves, and T-wave inversions in leads V_1-6_ and III. Echocardiography revealed right ventricular (RV) dilatation (RV outflow tract diameter/body surface area: 22.9 mm/m^2^) and reduced RV function (fractional area change: 18.0%). Cardiac magnetic resonance imaging confirmed RV dysfunction (ejection fraction [EF]: 9.7%) and left ventricular (LV) involvement (EF: 48.9%). Genetic testing identified compound heterozygous *DSG2* variants (p.Arg119* and p. Arg292Cys) and an *MYBPC3* variant (p.Arg820Gln). The patient remained asymptomatic until age 10.5 years, when she developed heart failure requiring hospitalization. Imaging revealed severe biventricular dilatation (LV end-diastolic volume index: 149.5 mL/m^2^; RV end-diastolic volume index: 255.9 mL/m^2^) and biventricular dysfunction (LVEF: 9.5%; RVEF: 9.7%). Despite medical management, the patient’s condition progressively worsened, and she was deemed eligible for heart transplantation.

**Discussion:**

This case illustrates the potential for severe pediatric ACM associated with compound heterozygous *DSG2* variants and a *MYBPC3* variant. The *DSG2* variants likely played a primary role disease pathogenesis, while the *MYBPC3* variant may have exacerbated the phenotype. The coexistence of desmosomal and sarcomeric gene variants is rare in cardiomyopathies, making genotype-phenotype correlations complex. Further research is needed to elucidate the interplay between these genetic factors.

**Conclusion:**

This case underscores the genetic heterogeneity and phenotypic variability in inherited cardiomyopathies. It emphasizes the importance of comprehensive genetic testing and close monitoring of affected individuals and their families.

## 1 Introduction

Desmoglein-2 (DSG2), a desmosomal cadherin protein encoded by the *DSG2* gene (Online Mendelian Inheritance in Man [OMIM] #125671), is critical for maintaining the structural integrity of cardiac desmosomes and intercalated discs. Pathogenic *DSG2* variants are a major genetic cause of arrhythmogenic cardiomyopathy (ACM), also known as arrhythmogenic right ventricular cardiomyopathy/dysplasia (ARVC/D). This inherited cardiac disorder is characterized by progressive fibrofatty replacement of the myocardium, leading to ventricular dilatation and, ultimately, heart failure (Basso et al., 2009). ACM is associated with an increased risk of life-threatening ventricular arrhythmias and sudden cardiac death (SCD) (Del Duca et al., 2024). The prevalence of *DSG2* pathogenic variants in patients with ACM varies across cohorts, ranging 6%–11% (Pilichou et al., 2006; Christensen et al., 2010; Jorda et al., 2022), but is notably higher in Japanese populations, reaching 31% (Wada et al., 2017). These variants often lead to reduced protein levels or impaired function, disrupting adhesion, binding, and signaling by altering interactions with partner proteins (Schinner et al., 2020; Hawthorne et al., 2021). Studies indicate that biallelic *DSG2* variants result in ACM phenotypes with 100% penetrance (Chen et al., 2019), whereas heterozygous *DSG2* variant carriers typically remain unaffected or exhibit only mild ACM-related symptoms in approximately 25% of relatives. This suggests that heterozygous carriers typically rarely develop the full ACM phenotype. The advent of next-generation sequencing has enhanced the detection of autosomal recessive ACM cases, particularly in individuals with compound heterozygous or homozygous *DSG2* variants (Qadri et al., 2017; Lin et al., 2018). Collectively, these findings strongly support that ACM due to *DSG2* variants follows an autosomal recessive inheritance pattern, requiring biallelic variants for the complete clinical phenotype to manifest.

Myosin-binding protein C, cardiac-type (MyBP-C), is a key structural component of the sarcomere encoded by the *MYBPC3* gene (OMIM # 600958). It is crucial in regulating cardiac muscle contraction (Topriceanu et al., 2024). The *MYPC3* gene is widely recognized as a major genetic cause of inherited hypertrophic cardiomyopathy (HCM), an autosomal dominant disorder and a significant contributor to SCD, particularly among adolescents and young adults (Marian and Braunwald, 2017). Although less frequently reported, *MYBPC3* variants have also been implicated in the etiology of ARVC (Choung et al., 2017; Murray et al., 2018).

ACM typically manifests during or after adolescence, and is rare in childhood. Even in genetically predisposed individuals, diagnostic criteria may not be met in early life (Corrado et al., 2017). While no direct evidence supports *MYBPC3* variants as phenotypic modifiers in ACM, we report the case of a 6-year-old girl carrying compound heterozygous *DSG2* variants (p.Arg119* and p. Arg292Cys, a desmosomal gene) and a *MYBPC3* variant (p.Arg820Gln, a sarcomere gene). Despite ACM’s usual late onset, this proband fulfilled the 2010 ARVC Revised Task Force Criteria (Marcus et al., 2010) at age 6. This case suggests that multiple pathogenic variants, including those in non-desmosomal genes such as *MYBPC3*, may contribute to a more severe ACM phenotype.

## 2 Case description

**FIGURE 1:**
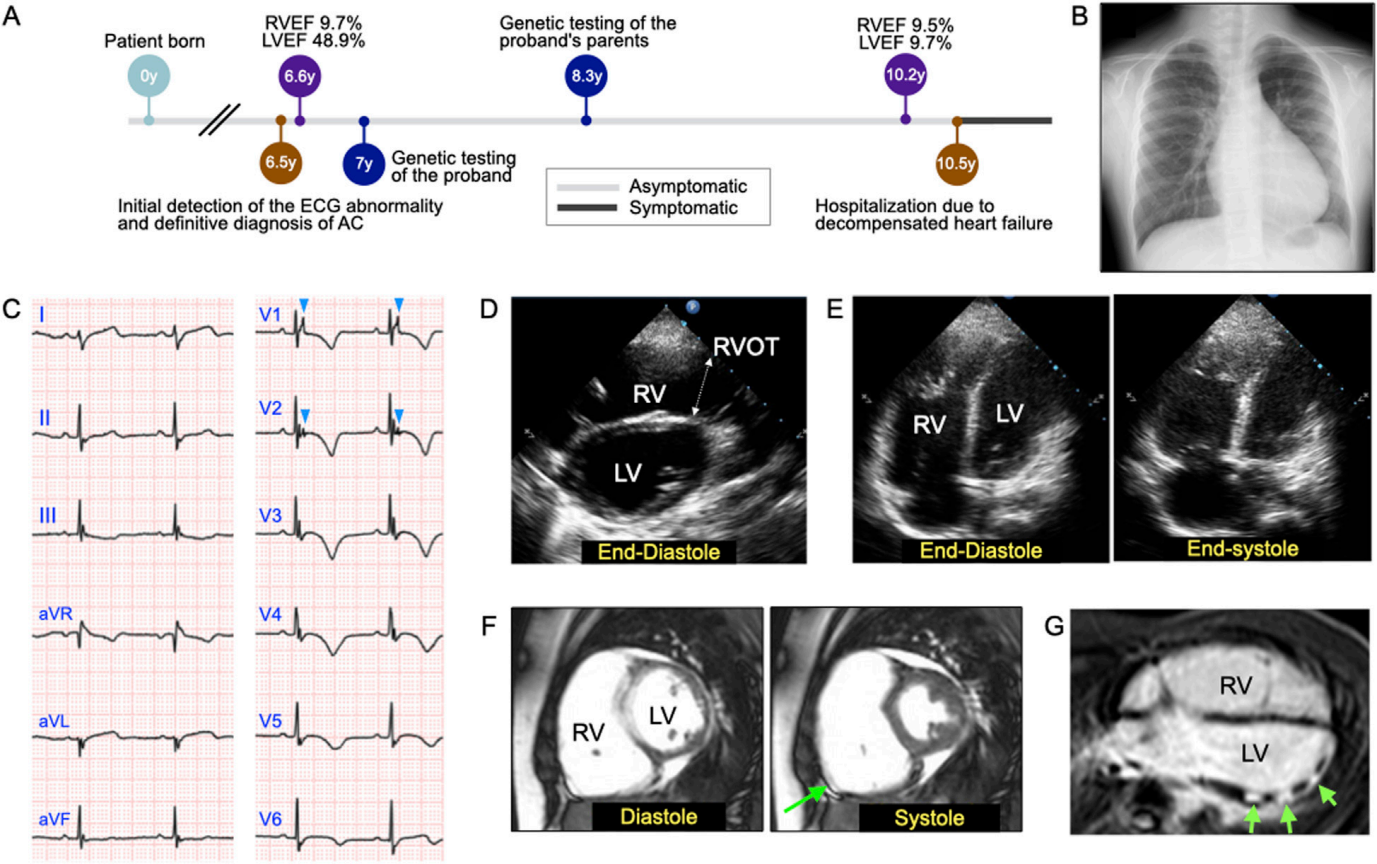
Objective findings at the initial examination of the 6-year-old patient with arrhythmogenic cardiomyopathy (ACM) and dilated cardiomyopathy (DCM). **(A)** Timeline of clinical investigations and disease-related events. **(B)** Chest radiograph revealing the protrusion of the right second and left fourth arches. **(C)** Electrocardiogram showing epsilon waves and T-wave inversion in leads V_1-6_ and lead III. The light blue arrowheads in leads V_1-2_ indicate epsilon waves. **(D)** Two-dimensional (2D) echocardiographic image acquired in the parasternal long-axis view at end-diastole. Echocardiography confirmed reduced contraction, mainly in the right ventricular outflow tract (RVOT), which measured 2.44 cm. RV, right ventricle; LV, left ventricle. **(E)** Representative 2D echocardiographic images acquired in the apical four-chamber view at end-diastole (left) and end-systole (right), which were used to calculate the RV fractional area change, were found to be 18.0%. **(F)** Cardiac magnetic resonance imaging (MRI) demonstrated dyskinesis of the RV base (green arrow), with an RV end-diastolic volume index (RVEDVI) of 139.9 mL/m^2^, RV ejection fraction (RVEF) of 9.7%, and a LV ejection fraction (LVEF) of 48.9%. **(G)** Delayed enhancement MRI scan acquired in the horizontal long-axis view, revealing patchy late gadolinium enhancement in the mid-to-apical region of the left ventricular anterolateral wall (green arrows).

A 6-year-old asymptomatic girl was referred to her pediatrician after an abnormal electrocardiogram (ECG) finding was observed during Japan’s mandatory first-grade health screening at school. The ECG revealed a right-axis deviation, although she had no clinical symptoms such as chest pain, dyspnea, or syncope. A subsequent echocardiogram at another facility identified right ventricular (RV) enlargement, prompting referral to our center for further evaluation and management (Figure 1A). At 6.5 years of age, initial assessment included chest radiography, which revealed protrusion of the right second and left fourth arches (Figure 1B). Electrocardiography showed epsilon waves and negative T waves in leads V_1-6_ and III (Figure 1C). Echocardiography confirmed thinning of the RV wall, reduced contraction of the RV outflow tract, and dyskinesis of the RV base. The RV outflow tract diameter in the parasternal long-axis view, adjusted for body surface area (PLAX/BSA), measured 22.9 mm/m^2^ (Figure 1D). Apical four-chamber echocardiography showed RV end-diastolic and end-systolic areas of 22.2 cm^2^ and 18.2 cm^2^, respectively, yielding a fractional area change (FAC) of 18.0% (Figure 1E). Cardiac magnetic resonance imaging (MRI) further confirmed dyskinesis of the RV base, with an RV ejection fraction (RVEF) of 9.7% and a left ventricular ejection fraction (LVEF) of 48.9% (Figure 1F). The RV end-diastolic volume indexed to body surface area (RVEDV/BSA) was 139.9 mL/m^2^, exceeding the normal reference range for females (≤100 mL/m^2^). Delayed enhancement MRI in the horizontal long-axis view demonstrated patchy late gadolinium enhancement in the mid-to-apical region of the left ventricular anterolateral wall (Figures 1F, G; green arrows). At this stage, the patient fulfilled at least two major criteria of the 2010 Task Force Criteria for ARVC (Marcus et al., 2010), confirming a clinical diagnosis of ACM. Given the diagnosis, immediate physical activity restrictions were advised, including avoidance of moderate- and high-intensity exercise typical for her age group.

**FIGURE 2:**
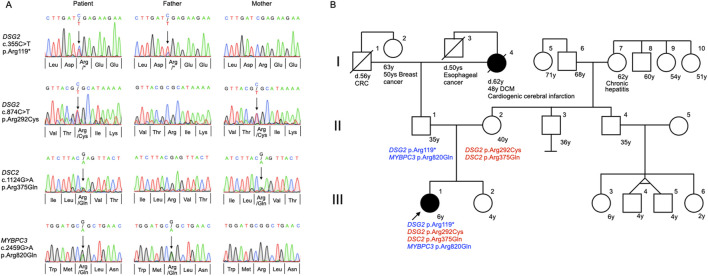
Trio analysis for genetic variants and pedigree analysis. **(A)** Direct Sanger sequencing confirmed the inheritance of variants in desmoglein-2 (*DSG2*), desmocollin-2 (*DSC2*), and myosin-binding protein C (*MYBPC3*) genes in the proband and her parents. **(B)** Family pedigree analysis of the 6-year-old proband (III-1) demonstrating compound heterozygosity for *DSG2* p. Arg119* and *DSG2* p. Arg292Cys variants, with an additional heterozygous *MYBPC3* p. Arg820Gln variant. The proband inherited the *DSG2* p. Arg119* and *MYBPC3* p. Arg820Gln variants paternally (II-2), while the *DSG2* p. Arg292Cys variant was transmitted maternally (II-2). The *DSC2* p. Arg375Gln variant, whose pathogenic significance remains unclear, was inherited from the mother. The proband’s paternal grandmother (I-4) was diagnosed with dilated cardiomyopathy (DCM) at the age of 48 and succumbed to a cardiogenic cerebral infarction at the age of 62. The arrow indicates the index patient. Solid black symbols in the pedigree denote individuals with a history of cardiac disease. CRC, colorectal cancer.

Following genetic counseling for the patient and her parents, a peripheral blood sample was sent to Shiga University of Medical Science for genetic testing of ACM-associated genes. At age 7, the patient underwent genetic profiling using a custom next-generation sequencing (NGS) panel (HaloPlex HS, Agilent Technologies, Santa Clara, CA, USA) targeting 61 genes linked to cardiac arrhythmias (Supplementary Table S1) (Fukuyama et al., 2023; Shimamoto et al., 2024). Initial sequencing was performed on an Illumina MiSeq platform (Illumina, San Diego, CA, USA), with variant analysis conducted using SureCall software (Agilent Technologies). Identified variants were validated via Sanger sequencing. Genetic analysis revealed two pathogenic *DSG2* variants (NM_001943.5:c.874C>T, p. Arg292Cys, rs770921270 and c.355C>T, p. Arg119*, rs753052874), along with variants in desmocollin-2 (*DSC2*) (NM_024422.6:c.1124G>A, p. Arg375Gln, rs770412621), and *MYBPC3* (NM_000256.3:c.2459G>A, p. Arg820Gln, rs2856655) (Table 1). Familial genetic analysis showed that the father carried *DSG2* p. Arg119* and *MYBPC3* p. Arg820Gln, while the mother carried *DSG2* p. Arg292Cys and *DSC2* p. Arg375Gln (Figure 2A). The proband inherited both *DSG2* variants in a compound heterozygous state (Figure 2B). The *DSC2* p. Arg375Gln is a missense variant, which results in arginine-to-glutamine substitution at codon 375. Given its similar biochemical properties, its pathogenicity remains uncertain. This variant has not been previously linked to ACM, and *in silico* predictions yielded inconclusive results (Supplementary Table S2). Consequently, it is classified as a variant of uncertain significance and is unlikely to contribute to disease pathogenesis in this case. Based on genetic findings, this case meets the criteria for definite biventricular ACM, as defined by the more recently proposed Padua criteria (Corrado et al., 2020; Graziano et al., 2022) (Supplementary Table S3).

**TABLE 1:** Summary of the variants detected in the proband and family members.

Gene	References	Location (cytogenetic)	Location (GRCh38)	Exon	Nucleotide change	Protein change	SNP ID	ClinVar Classification*	Allele frequency (60KJPN)	Patient	Mother	Father
*DSG2*	NM_001943.5	18q12.1	18: 31520941	exon4	c.355C>T	p.Arg119*	rs753052874	Conflicting classifications of pathogenicity**	0.000067	+	-	+
*DSG2*	NM_001943.5	18q12.1	18: 31524748	exon8	c.874C>T	p.Arg292Cys	rs770921270	Uncertain significance	0.002561	+	+	-
*DSC2*	NM_024422.6	18q12.1	18: 31082377	exon9	c.1124G>A	p.Arg375Gln	rs770412621	Uncertain significance	0.00000***	+	+	-
*MYBPC3*	NM_000256.3	11p11.2	11: 47337534	exon25	c.2459G>A	p.Arg820Gln	rs2856655	Pathogenic/Likely pathogenic	0.000242	+	-	+

*accessed on 5 December 2024, **Pathogenic (3), Uncertain significance (2); ***gnomAD-Exomes, Asian; *DSG2,* desmoglein-2; *DSC2,* desmocollin-2; *MYBPC3,* myosin-binding protein C; SNP, single nucleotide polymorphism.

Family history revealed that the proband (III-1), her younger sister (III-2), and both parents (II-1, II-2) were asymptomatic. She had no notable medical history aside from her cardiac condition. Her paternal grandmother (I-4) was diagnosed with dilated cardiomyopathy (DCM) at the age of 48 and died at 62 from a cardioembolic stroke. No cardiac disease was reported among the proband’s second-degree relatives (Figure 2B).

## 3 Follow-up

**FIGURE 3:**
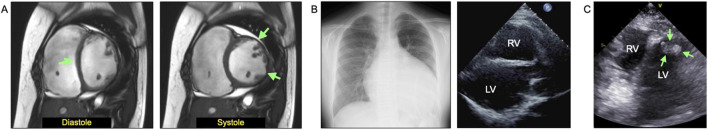
Clinical findings from the most recent pre- or post-admission examinations at 10.5 years of age (4 years after diagnosis) when the patient developed heart failure. **(A)** Cardiac magnetic resonance imaging (MRI) performed 17 days before admission showed marked increases in ventricular volume and decreased contractility not only in the right ventricle (RV) but also in the left ventricle (LV), with a RV ejection fraction (RVEF) of 9.7% and a LV ejection fraction (LVEF) of 9.5%. Septal displacement (diastole, green arrow) and aneurysmal thinning and dyskinesis of the anterolateral wall of the LV (systole, green arrow) were observed. **(B)** Chest radiograph at admission shows marked cardiomegaly, and echocardiography reveals bilateral ventricular dilatation and severe hypokinesis. **(C)** Left ventricular apical thrombus (17 × 11 mm) confirmed 3 weeks after admission.

The patient remained asymptomatic for heart failure throughout childhood, largely due to strict exercise restrictions. However, at 10.5 years of age, she developed fatigue and bilateral lower leg edema, leading to hospitalization for heart failure treatment. Cardiac MRI performed 17 days before admission revealed significant ventricular deterioration, with markedly increased ventricular volumes (LV end-diastolic volume index: 149.5 mL/m^2^; RV end-diastolic volume index: 255.9 mL/m^2^) and severely reduced contractility (LVEF: 9.7%; RVEF: 9.5%). Detailed cardiac function measurements are presented in Supplementary Table S4. Additional findings included septal displacement during diastole, aneurysmal thinning, and dyskinesis of the LV anterolateral wall during systole (Figure 3A). Upon admission, chest radiography revealed marked cardiomegaly, while echocardiography confirmed bilateral ventricular dilatation and severe hypokinesis (Figure 3B). Treatment was initiated immediately, including continuous intravenous infusion of human atrial natriuretic peptide alongside oral angiotensin-converting enzyme inhibitors (ACEi), mineralocorticoid receptor antagonists (MRA), and loop diuretics (LD). Despite no significant improvements in cardiac MRI findings at discharge (LVEF: 13.4%; RVEF: 9.1%), clinical symptoms improved. Serum brain natriuretic peptide levels decreased from 1,586 pg/mL at admission to 382 pg/mL at discharge, and body weight reduced from 50.15 kg to 46.2 kg.

Approximately 3 weeks after admission, a left ventricular apical thrombus (17 × 11 mm) was detected (Figure 3C), which had been absent at admission. The patient was started on continuous intravenous heparin, followed by oral warfarin, leading to a reduction in thrombus size (7 × 5 mm by discharge). During hospitalization, continuous ECG monitoring revealed 3–4 episodes of slow ventricular tachycardia, and no other significant arrhythmias were detected. The proband’s parents had no history of arrhythmia. On day 4 of admission, oral amiodarone therapy was initiated, effectively suppressing premature ventricular contractions. The patient was discharged approximately 2.4 months after admission, with symptom resolution. Post-discharge, she remained asymptomatic under oral treatment with ACEi, MRA, LD, amiodarone, biaspirin, and warfarin. The ventricular thrombus continued to regress with anticoagulation therapy, and echocardiographic examinations consistently showed no outflow tract obstruction throughout the clinical course. The patient was evaluated and deemed eligible for heart transplantation, and registration at a transplantation facility in Japan is currently in progress.

## 4 Discussion

In the present case, three germline variants in desmosomal genes (*DSG2* p. Arg292Cys, *DSG2* p. Arg119*, and *DSC2* p. Arg375Gln) and one variant in a sarcomeric gene (*MYBPC3* p. Arg820Gln) were identified. Among these, *DSG2* p. Arg292Cys has been previously reported in Japanese patients with ACM (Wada et al., 2017). *DSG2* encodes a cadherin-family protein involved in calcium-dependent cell-cell adhesion. It interacts with desmocollin-2 to form desmosomes, by forming highly ordered adhesion cluster structures, essential for robust intercellular adhesion (Tariq et al., 2015; Goz et al., 2024). *DSG2* Arg292 is located in the extracellular cadherin domain and is predicted to form hydrogen bonds with at least three neighboring residues, stabilizing its beta-strand structure and maintaining its function (Supplementary Figure S1). The *DSG2* p. Arg119* variant introduces a premature stop codon in exon 4, likely leading to nonsense-mediated mRNA decay rather than protein truncation. This is due to the proximity of the premature termination codon to the 5′end of the transcript (Vallverdu-Prats et al., 2022). Given its expected loss-of-function mechanism, the variant was classified as disease-causing in the present case. Familial genetic analysis confirmed that the proband carried both *DSG2* variants in a biallelic state, with no detectable wild-type *DSG2* allele. The *MYBPC3* gene encodes the cMyBP-C protein, a key sarcomeric component that interacts with various sarcomeric proteins such as, myosin, titin, and actin to regulate thick-thin filament interaction (Heling et al., 2020). The *MYBPC3* p. Arg820Gln variant, frequently identified in Japanese patients with HCM (Nakashima et al., 2020; Hiruma et al., 2024), may play a role in modifying the cardiac phenotype. Arg820 is located within the fibronectin type 3-like domain (C6) of the cMyBP-C. While nuclear magnetic resonance spectroscopy and small-angle X-ray scattering indicate no direct structural disruption (Nadvi et al., 2016), the variant has been reported to induce haploinsufficiency via mRNA destabilization or reduced protein stability (Pearce et al., 2024). In summary, two *DSG2* variants (*DSG2* p. Arg292Cys and *DSG2* p. Arg119*) and one *MYBPC3* variant (*MYBPC3* p. Arg820Gln) were strongly implicated in the pathogenesis of ACM in this case. However, the pathogenic significance of *DSC2* p. Arg375Gln remains uncertain.

*DSG2* variants are more frequently observed in Asian populations compared to Caucasians (Ohno, 2016). In ACM cohorts primarily comprising Caucasian individuals, *PKP2* gene variants are the most prevalent, while *DSG2* variants are underrepresented (Paldino et al., 2022). In contrast, a Japanese ACM cohort revealed that *DSG2* variants were the most common (48%), followed by *PKP2* variants (38%) (Wada et al., 2017). Interestingly, while probands in the Japanese study exhibited a high incidence of life-threatening cardiac events, their heterozygous family members rarely developed ACM-related symptoms. These findings strongly suggest that most *DSG2*-related ACM cases follow an autosomal recessive inheritance pattern, requiring biallelic variants for disease manifestation. This hypothesis is further reinforced by the present case, in which the patient’s parents, both heterozygous *DSG2* variant carriers, remained phenotypically unaffected. Understanding this inheritance pattern is crucial for accurate genetic counseling, particularly in Japan, where *DSG2* variants are more prevalent compared to that in Caucasian populations.

In the present case, compound heterozygous pathogenic variants in desmosomal genes were identified, along with a heterozygous variant in the sarcomeric gene *MYBPC3*. Pathogenic *MYBPC3* variants are a well-documented cause of HCM (Tudurachi et al., 2023; Abbas et al., 2024). A subset of HCM cases progresses to a dilated phase, characterized by LV thinning and dilatations, often mimicking DCM (Marstrand et al., 2020; Ishihara et al., 2023). Recent familial HCM cohort studies have identified *MYBPC3* pathogenic variants as the most common cause, with truncating variants accounting for 91% of *MYBPC3*-related cases (Helms et al., 2020). Experimental studies using differential scanning fluorimetry and microscale thermophoresis have shown that the p. Arg820Gln variant does not significantly affect the thermal stability, myosin binding affinity, or actomyosin ATPase activity of cMyBP-C (Pearce et al., 2024). However, evidence suggests that the p. Arg820Gln may impair protein stability in eukaryotic cells (Pearce et al., 2024). Additional studies propose that the p. Arg820Gln variant may disrupt protein-protein interactions (Nadvi et al., 2016) or cause mRNA destabilization (Suay-Corredera et al., 2021). Furthermore, missense *MYBPC3* variants may influence calcium handling within cardiomyocytes (Pioner et al., 2023). Given these findings, definitive conclusions regarding the contribution of *MYBPC3* p. Arg820Gln to the proband’s condition remain challenging. While ClinVar classifies this variant as pathogenic/likely pathogenic, reliable data on age-dependent penetrance in heterozygous carriers are currently lacking. Notably, the proband’s father (II-1), who carries the p. Arg820Gln variant, remains asymptomatic, suggesting that this variant alone is unlikely to cause early-onset HCM. Additionally, there is no direct experimental evidence supporting the hypothesis that the *MYBPC3* p. R820Q variant increases mechanical stress on the sarcomere. However, the potential contribution of this variant to disease severity cannot be excluded. The proband in this case report presented with heart failure as the initial symptom and no severe arrhythmias were recorded throughout hospitalization, including during continuous ECG monitoring. Based on these observations, it can be concluded that heart failure, rather than a primary arrhythmia syndrome, was the predominant clinical manifestation in this proband, likely influenced by the *MYBPC3* variant.

Reports of cardiomyopathies arising from the coinheritance of desmosomal and sarcomeric gene mutations are exceedingly rare. De Bortoli et al. (2017) reported cases involving concurrent mutations in the desmosomal gene *DSP* and the sarcomeric gene *MYBPC3*, as well as in catenin alpha 3 (*CTTNA3*) and myosin heavy chain 7 (*MYH7*). Among four clinically evaluated individuals with *DSP* and *MYBPC3* mutations, two were diagnosed with ACM and two with HCM, indicating variability in clinical expression. These inter-individual differences may be attributed to age- and sex-related incomplete penetrance of the co-inherited mutations, complicating the determination of each variant’s pathogenic role. Sakamoto et al. (2019) reported a 47-year-old woman with left-dominant arrhythmogenic cardiomyopathy who harbored mutations in *DSP* and *MYBPC3*, including the same p. Arg820Gln variant identified in the present report. The case featured mild LV dilatation with global and regional systolic dysfunction, preserved RV volume and contraction, and fatty replacement of the LV wall. In contrast, Yang et al. (2021) described a 35-year-old woman with *DSP* and *MYBPC3* co-mutations, who presented with coarctation of the aorta, HCM, and supraventricular tachycardia, but without notable RV dysfunction or imaging abnormalities. To our knowledge, no previous reports have described cardiomyopathy associated with compound heterozygous or homozygous *DSG2* variants coexisting with a pathogenic *MYBPC3*variant, as seen in the present case. Genetic testing of the proband (III-1) family suggests that the combination of two *DSG2* variants and one *MYBPC3* variant may have synergistically contributed to the severe phenotype. However, the precise pathogenic contribution of each variant remains uncertain. While their co-occurrence may explain the proband’s clinical severity, the influence of additional genetic or environmental factors cannot be excluded. The genotype of the unaffected second child (III-2) could offer valuable insights, but due to ethical considerations, genetic testing has not been performed. Further research, including functional studies and larger cohort analyses, is needed to clarify potential variant interactions and their individual contributions to the disease pathogenesis. Until more conclusive evidence emerges, the hypothesis of a synergistic effect remains speculative. These findings highlight the genetic heterogeneity and phenotypic variability of inherited cardiomyopathies. Factors such as incomplete penetrance, gene-gene interactions, and environmental influences complicate the interpretation of genotype-phenotype correlations, making genetic counseling particularly challenging in these cases.

This study employed a targeted 61-gene panel focused on inherited arrhythmias and cardiomyopathies. While effective in identifying key variants, this approach has limitations. Targeted panels may miss novel or rare variants outside the selected genes. Although all detected variants were confirmed by Sanger sequencing, whole-genome sequencing, epigenomic analysis, and protein-level investigations were not performed. Despite these limitations, our findings provide strong evidence supporting the pathogenic role of the identified variants in the patient’s condition. Future studies incorporating more comprehensive approaches, such as whole-genome sequencing and molecular analyses, could further clarify the genetic and molecular mechanisms underlying the disease phenotype.

This report describes the case of a young girl with a severe form of ACM complicated by DCM. Genetic testing revealed compound heterozygous pathogenic variants in the *DSG2* gene and a pathogenic variant in the *MYBPC3* gene. Despite her young age of 6 years, she faces significant challenges due to these genetic conditions. Looking ahead, with advancements in gene therapy, molecular-targeted therapies and xenotransplantation hold promise for transforming the management of inherited cardiomyopathies in the coming decades.

## Data Availability

The original contributions presented in the study are included in the article/Supplementary Material, further inquiries can be directed to the corresponding authors.
