# Supplementary material for: Case report: Severe arrhythmogenic cardiomyopathy in a young girl with compound heterozygous DSG2 and MYBPC3 variants with a 6-year follow-up
Source: Front Genet. 2025 Mar 6;16:1545561. doi: 10.3389/fgene.2025.1545561 (PMC11922858; doi:10.3389/fgene.2025.1545561)
Supplement: Supplementary file 1 [file Table1.docx]

Supplementary Material

# Supplementary Tables

**Supplementary Table S1.** Target genes of the arrhythmia panel used in this study

| *AKAP9* | *CASQ2* | *GJA5* | *KCNE3* | *KCNN2* | *SCN10A* | *SNTA1* |
| --- | --- | --- | --- | --- | --- | --- |
| *ANK2* | *CAV3* | *GJC1* | *KCNE4* | *KCNQ1* | *SCN1B* | *TBX5* |
| *CACNA1C* | *CHRM2* | *GLA* | *KCNE5* | *LMNA* | *SCN2B* | *TCAP* |
| *CACNA2D1* | *DES* | *HCN4* | *KCNH2* | *MYBPC3* | *SCN3B* | *TECRL* |
| *CACNB2* | *DSC2* | *JUP* | *KCNIP2* | *MYH6* | *SCN4B* | *TMEM168* |
| *CALM1* | *DSG2* | *KCNA5* | *KCNJ2* | *NKX2-5* | *SCN5A* | *TMEM43* |
| *CALM2* | *DSP* | *KCND3* | *KCNJ3* | *PKP2* | *SLC4A3* | *TRDN* |
| *CALM3* | *FLNC* | *KCNE1* | *KCNJ5* | *PRKAG2* | *SLC8A1* | *TRPM4* |
| *CAMK2D* | *GJA1* | *KCNE2* | *KCNJ8* | *RYR2* |  |  |

**Supplementary Table S2.** *In silico* analysis of the desmocollin-2 (*DSC2*) p.Arg375Gln variant

| **Prediction Tool** | **Prediction Results** | **Reference** |
| --- | --- | --- |
| SIFT | Tolerated | Ng and Henikoff, 2001 [PMID: 11337480] |
| PolyPhen-2 | Probably Damaging | Adzhubei et al., 2010 [PMID: 20354512] |
| Align-GVGD | Class C35 | Tavtigian et al., 2006 [PMID: 16014699] |
| AFFIPred | Benign | Janes et al., 2024 [PMID: 38854010] |

**Supplementary Table S3.** Classification according to the Padua criteria

|  | **Right ventricle** | **Left ventricle** |
| --- | --- | --- |
| I. Morpho-functional ventricular abnormalities | Major (+) | Minor (+) |
| II. Structural myocardial abnormalities | – | Major (+) |
| III. ECG repolarization abnormalities | – | Minor (+) |
| IV. ECG depolarization abnormalities | Minor (+) | – |
| V. Ventricular arrhythmias | – | – |
| VI. Family history/genetics | Major (+) | |
| The final diagnosis | definite biventricular ACM | |

**Supplementary Table S4.** Summary of cardiac function measurements

|  |  | Age 6 | Age 10.5 |
| --- | --- | --- | --- |
|  |  |  |  |
| ECG | Heart rate | 57 bpm | 93 bpm |
|  | Leads showing epsilon waves | V_1_-V_4_, III | V_1_-V_4_ |
|  | Leads showing negative T waves | V_1_-V_6_, III, aVR | V_1_-V_6_, III |
|  | QRS axis | 102° | 120° |
|  |  |  |  |
| Echocardiography | Heart rate | n/r | 84 bpm |
|  | RV end-diastolic area^#^ | 22.2 cm^2^ | 40.3 cm^2^ |
|  | RV end-systolic area^#^ | 18.2 cm^2^ | 35.9 cm^2^ |
|  | RV fractional area change | 18% | 11% |
|  | PLAX/BSA | 22.9 mm/m^2^ | 16.7 mm/m^2^ |
|  |  |  |  |
| MRI | Heart rate | 70 bpm | 77 bpm |
|  | RV end-diastolic volume | 142.4 mL | 358.1 mL |
|  | RV end-systolic volume | 128.6 mL | 324.1 mL |
|  | RV stroke volume | 13.8 mL | 34.1 mL |
|  | RV ejection fraction | 9.7% | 9.5% |
|  | RV cardiac output | 0.97 L/min | 2.62 L/min |
|  | RV end-diastolic volume index | 139.9 mL/m^2^ | 255.9 mL/m^2^ |
|  | RV end-systolic volume index | 126.4 mL/m^2^ | 231.6 mL/m^2^ |
|  | RV stroke volume index | 13.6 mL/m^2^ | 24.4 mL/m^2^ |
|  | RV cardiac index | 0.95 L/min/m^2^ | 1.87 L/min/m^2^ |
|  | LV end-diastolic volume | 66.2 mL | 209.2 mL |
|  | LV end-systolic volume | 33.8 mL | 188.9 mL |
|  | LV stroke volume | 32.4 mL | 20.3 mL |
|  | LV ejection fraction | 48.9% | 9.7% |
|  | LV cardiac output | 2.27 L/min | 1.56 L/min |
|  | LV end-diastolic volume index | 65.0 mL/m^2^ | 149.5 mL/m^2^ |
|  | LV end-systolic volume index | 33.2 mL/m^2^ | 135 mL/m^2^ |
|  | LV stroke volume index | 31.8 mL/m^2^ | 14.5 mL/m^2^ |
|  | LV cardiac index | 2.23% | 1.12% |
|  | LV mass | 36.5 g | 90.9 g |
|  | LV mass index | 35.9 g/m^2^ | 43.5 g/m^2^ |
|  |  |  |  |

n/r, Not recorded; ^#^measured via apical four-chamber echocardiography. RV, right ventricular; LV, left ventricular; PLAX/BSA, RV outflow tract diameter in the parasternal long-axis view adjusted for body surface area.


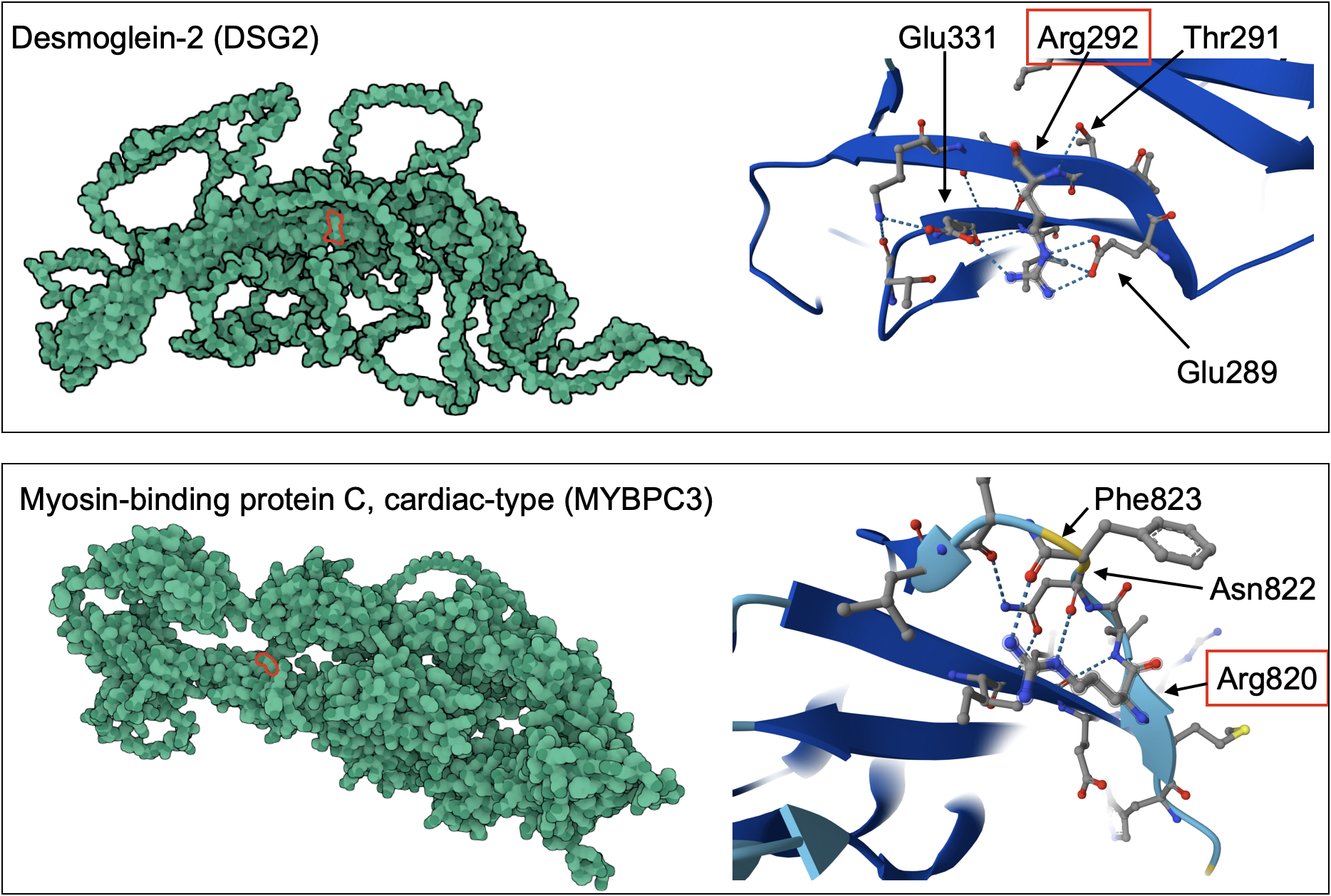


**Supplementary Figure S1**. Structural models of proteins encoded by the *DSG2* and *MYBPC3* genes, illustrating the locations of missense variants. The right panel depicts the overall molecular structure, with the amino acids harboring missense variants highlighted by red circles. The left panel provides a magnified view of the variant sites. In the magnified view, neighboring amino acid residues that form hydrogen bonds with the wild-type amino acids are labeled. The structural predictions were generated using the AlphaFold Protein Structure Database (https://alphafold.ebi.ac.uk/), developed by EMBL-EBI (accessed on January 7, 2025). *DSG2* Arg292 is located within a beta strand in the extracellular topological domain, while *MYBPC3* Arg820 is positioned in the fibronectin type-III like domain. *DSG2* Arg292 and *MYBPC3* Arg820 are predicted to contribute to the structural integrity by forming hydrogen bonds with three and two neighboring amino acids, respectively.
